# Carbon footprint awareness of nursing students: a qualitative study

**DOI:** 10.1186/s12912-025-03021-z

**Published:** 2025-03-31

**Authors:** Mağfiret Kaşıkçı, Yeşim Bağcı, Zeynep Yıldırım, Ulviye Aydan Nacak

**Affiliations:** 1 Departman of Fundamental of Nursing, Faculty of Nursing, Ataturk University, Erzurum, Turkey; 2 Department of Nursing, Faculty of Health Sciences, Ardahan University, Ardahan, Turkey

**Keywords:** Nurse, Student, Carbon footprint, Awareness, Qualitative study

## Abstract

**Backround:**

The increasing impact of climate change on public and environmental health underscores the importance of understanding carbon footprints and adopting sustainable practices. Nursing students play a critical role in promoting environmental awareness and integrating eco-friendly practices into healthcare delivery. This study aims to assess nursing students’ awareness of the ‘Carbon Footprint’ and their suggestions for reducing their carbon footprint.

**Design:**

Qualitative study.

**Methods:**

Twelve fourth-year nursing students enrolled in the “Public Health Nursing” course within the nursing department from a university located in eastern Turkey participated in this study. Twelve face-to-face interviews were conducted. Transcripts were subjected to structured qualitative content analysis.

**Results:**

As a result of the analysis of the data obtained from the participants, two main themes as ‘Perceived Impacts of Carbon Footprint’ and ‘Carbon Footprint Reduction Strategies’, four sub-themes as ‘Perceived Impact on Human Health’, ‘Perceived Impact on Environmental Health’, ‘Recommended Individual Strategies’ and ‘Recommended Institutional-Level Strategies’ and eighteen codes in total were created.

**Conclusion:**

This study revealed that nursing students possess a fundamental awareness of the carbon footprint and its implications for human and environmental health. Integrating comprehensive environmental education into nursing curricula can empower future healthcare professionals to adopt and advocate for sustainable practices in both personal and professional domains.

## Introduction

The United Nations’ Sustainable Development Goal 13, entitled ‘Climate Action’, emphasises the necessity of urgent action to combat climate change. Climate change is regarded not only as an environmental problem but also a global crisis that has a direct impact on public health [1]. Environmental sustainability and the effects of climate change on the health of individuals are among the important issues of interest to the nursing profession. The theoretical foundations of nursing, which include the concept of environment, are of particular relevance in this context. The environment encompasses all the physical, biological, social and cultural domains in which individuals live and which directly impact their health [2]. In the domain of nursing practice, the interaction between the individual and their environment constitutes a pivotal focal point. Consequently, nurses consider environmental factors within the overarching process of health protection and improvement [3]. Florence Nightingale, the founder of modern nursing, was the first theorist to emphasise the effect of the environment on health. Nightingale emphasised and proved that improving the physical environment should be one of the most important struggles of nursing with her notes and statistics [4]. Also many nursing theorists have mentioned the effect and importance of the concept of environment on human health [5].

In recent years, the impact of the environment on health has become a more pressing global concern [6]. Of particular concern is the issue of carbon footprint, which has been identified as a primary contributor to environmental degradation and climate change [7]. The term ‘carbon footprint’ refers to the total amount of greenhouse gases released into the atmosphere as a result of the activities of individuals, communities or organisations [8, 9]. This is influenced by a multitude of factors, including energy use, transport, consumption habits and waste management [8]. Nevertheless, the reduction of carbon footprint is achievable through conscious choices made in daily life and at the institutional level [7]. It has been asserted that healthcare professionals, with a particular emphasis on nurses, should exhibit heightened environmental sensitivity and implement requisite precautionary measures [10]. The International Council of Nurses (ICN) has encouraged nurses and nursing associations to take action to reduce the negative impact of climate change on public health [11].

The field of nursing education encompasses not only the acquisition of professional knowledge and skills, but also the cultivation of sensitivity towards public health [12]. The enhancement of environmental awareness among nursing students has the potential to facilitate the implementation of environmentally sustainable policies within healthcare services in the future [13]. However, for this awareness-raising initiative to have a tangible impact on health policies, it is essential that environmental awareness and sustainability issues are adequately incorporated into the nursing education curriculum. In this context, it is critical to investigate the extent to which environmental sustainability and carbon footprint reduction issues are addressed in the nursing education process [14].

In the literature, it is noteworthy that the studies examining the level of knowledge and awareness of nursing students about carbon footprint are limited [15, 16]. Mohammed et al. [17], revealed that the issue of carbon footprint is not sufficiently addressed in the education process of health professionals and there are deficiencies in the development of environmental awareness. The aim of this study is to examine nursing students’ awareness of carbon footprint, their behaviours towards reducing carbon footprint in their daily lives and their attitudes towards this issue. In addition, by measuring the environmental awareness levels of nursing students, it aims to contribute to the vocational education process and to help the spread of environmental awareness in health services.

## Method

### Design

This study was conducted using a descriptive phenomenological design, which is a qualitative research method. The focus of phenomenological studies is to make sense of phenomena through the perspectives and life experiences of study participants. This methodological approach has been found to make significant contributions to both the scientific literature and the field of practice [10, 18, 19]. As this study is a qualitative study, Clinical trial number: not applicable.

### Settings and participants

The research was conducted between November 2024 and January 2025. The data were collected in December 2024. The study sample was obtained from a class of 60 fourth-year nursing students in the Nursing Department, with 12 students participating in the study. Within the scope of the sample of the study, it was aimed to interview the students taking the Public Health Nursing course. This course is given in the seventh semester in the relevant educational institution. A simple random number table was used in sample selection [20]. The inclusion criteria were to volunteer to participate in the study, to be over 18 years of age, to be a nursing student, to be a 4th year student and to have taken the ‘Public Health Nursing’ course. Accordingly, a total of twelve students, seven female and five male, volunteered to participate in the study and gave informed consent. First year students because they had not yet started their internship and 2nd and 3rd year students because they had not yet taken the ‘Public Health Nursing’ course were not included in the study. In determining the sample size, data collection was terminated when a sufficient amount of data was obtained and the saturation point was reached [19, 21]. The reason for the selection of students who have taken the Public Health Nursing course is that issues such as environmental health and carbon footprint are addressed within the scope of this course and these students have the level of knowledge that can provide data suitable for the purpose of the study. In addition, it was thought that the awareness of these students on the subject may be higher than other classes due to the course content.

### Data collection tools and features

The data were collected with ‘Demographic Information Form’ and ‘Semi-structured Interview Form’. The introductory form included information about the students’ gender, age, marital status, economic status, place of residence, participation in social and scientific activities, and carbon footprint. In the semi-structured interview form, students were asked six open-ended interview questions (see Table 1). The semi-structured questions were submitted to expert opinion (experts: a professor specialised in qualitative research in the department of basic education, a professor in the field of nursing, two doctoral faculty members in the field of nursing). In line with the feedback received in this process, the questions were re-evaluated and necessary arrangements were made. After the expert opinion was received, three students were interviewed for pilot application to examine the appropriateness of the interview questions and the semi-structured interview form was finalised in line with the feedback. Students who participated in the pilot study were not included in the study. The quotations made from the participants during the research were labelled as ‘Participant’ to protect anonymity in the text and each participant was assigned a number (e.g., P1). These numbers indicate which participant the quotations belong to and ensure traceability of the data. The assignment of participant numbers was done in order to protect the confidentiality of the participants’ identity information and to present the data in an ethical manner.

**Table 1:** Semi-structured interview questions

1. What are the measures you take to protect the environment in your daily life?
2. What do you think are the effects of single-use plastic products (straws, water bottles, etc.) on the environment?
3. How do you pay attention to waste management during clinical/field practices?
4. What do you know about the effects of carbon footprint on plant, animal species and ecosystems?
5. What do you think are the impacts of carbon footprint on human health?
6. In your opinion, if carbon footprint is recognised as a public health problem, what can be done to solve this problem?

### Data collection

Data were collected through face-to-face in-depth interviews. Since one of the inclusion criteria was to have taken the ‘Public Health Nursing’ course, data were collected after the last week of the fall term. Participants were informed about the scope and aims of the study. Before starting data collection with the main participant group, the interview programme was piloted with a fourth year nursing student who met the inclusion criteria. This pilot test allowed refinement of the interview questions and ensured clarity and comprehensiveness. Data were collected using an introductory information form and a semi-structured interview form. The introductory information form took an average of 2–3 min to complete and the interviews with the students took 20–25 min. In-depth interviews were conducted in the researcher’s room, which was quiet and well-lit so that individuals could express themselves uninterruptedly. As the interviewer, the researcher who was not attending their classes was selected (U.A.N.) so that the students would not experience grade anxiety and could express themselves more comfortably. Permission was obtained from the participants to record the interviews and a voice recorder was used during the interviews. The recordings were converted into a 13-page written text. Data collection was terminated when the data started to repeat.

### Data analysis

COREQ (Consolidated Criteria for Reporting Qualitative Research) reporting standards were followed to increase transparency and reliability in qualitative research [22]. For qualitative data analysis; all recorded interview data were transcribed without any changes. Socio-demographic variables of the students were analysed. The data of the study were analysed by content analysis (inductive) method. Data analysis was carried out by hand coding by the researchers. After the interviews were completed, the audio recordings were converted into written texts by the researchers. Written texts were checked by the researcher (Z.Y.). Written texts were analysed in 4 stages by content analysis method.

- Stage 1: Coding the data: The information obtained from each question of the interviews was analysed and divided into sections that form a meaningful whole. Codes were created one by one from these sections. The codes were transferred to the excel programme.
- Stage 2: Creation of themes: The codes obtained were brought together and analysed. Commonalities between these codes were tried to be found. Themes were created based on the codes categorised according to their scope and depth.
- Stage 3: Organising the data according to the codes and themes: The data were organised in a related way and thus it became easier to define and interpret the data according to certain phenomena. These definitions were included in the findings.
- Stage 4: Interpretation of the findings: After the findings defined based on the data were presented as they were, the comments and opinions of the researcher were included at the end of each finding.

The evaluation of the quantitative data of the students was expressed with numerical data after being presented in tables.

### Ethical considerations

Ethical approval was obtained from the Health and Sports Sciences Ethics Committee of Erzincan Binali Yıldırım University with the decision numbered 11/07 dated 29/11/2024. In addition, written permission was obtained from the institution where the data were collected. The study was conducted in accordance with the Declaration of Helsinki.

Detailed information was provided to the participating students about the purpose, methodology, and methods of the study. Participants were assured that participation in the study was voluntary and that they could withdraw from the study at any time without giving any reason. During the informed consent process, both written and verbal consent forms were presented to the participants. Students who refused to participate or did not allow audio recording were not included in the study. Interviews were conducted only with individuals who gave informed consent. All data were anonymized, preserving the confidentiality of the participants’ identities.

## Results

The analysis results obtained from the interviews conducted to examine nursing students’ awareness of carbon footprints and their suggestions are presented below.

**Table 2:** Demographic characteristics of nursing students (*N* = 12)

Variables	Category (*N*)
Gender	Female (*N* = 7) Male (*N* = 5)
Age	22–24
Income level	Middle (*N* = 12)
Residence	City (*N* = 10) District (*N* = 2)
Participation in Scientific Activities	Yes (*N* = 12)
Participation in Social Activities	Yes (*N* = 12)
Knowing the Concept of Carbon Footprint	Yes (*N* = 12)
Where did she/he learn about her carbon footprint?	Faculty (*N* = 3) Faculty/Social media (*N* = 2) Circle of friends (*N* = 1) Conference (*N* = 3) Faculty/ Circle of friends (*N* = 1) TV/ Social media (*N* = 1) Faculty/ Conference (*N* = 1)
Receiving training on carbon footprinting	Yes (*N* = 6) No (*N* = 6)
Self-Considered Environmentalist	Yes (*N* = 11) Undecided (*N* = 1)
Membership status of the Green Crescent club	Yes (*N* = 2) No (*N* = 10)

The ages of the students participating in the study range from twenty-two to twenty-four. Seven of the participants are female, and all belong to the middle-income group. Ten students reside in urban areas, and all reported participating in both scientific and social activities. All students indicated that they were familiar with the concept of carbon footprint; half of them stated that they learned about it through school courses, while the other half reported having received formal education on the topic. Eleven students described themselves as environmentally conscious, while ten were not members of the Green Crescent Club (Table 2).

**Table 3:** Themes, Sub-Themes, and codes obtained from interviews

Theme	Sub-Theme	Codes	Participant
Perceived Impacts of Carbon Footprint	Perceived Impact on Human Health	Affects the respiratory system	1,2,3,4,5,6,7,8,10,12
		Affects the circulatory system	4,6,8,10,12
		Reduces immunity	2,4,9
		Increases the risk of cancer	6,9,11
	Perceived Impact on Environmental Health	Soil pollution caused by plastic products	1,2,3,4,5,6,7,9,10,11,12
		Extinction of species	1,2,6,9,10
		Reduction in biodiversity	2,5,11
Carbon Footprint Reduction Strategies	Recommended Individual Strategies	Paying attention to waste management	1,2,3,4,5,6,7,8,9,10,11,12
		Not littering	1,2,3,4,5,6,8,9,10,11,12
		Sorting waste	1,2,4,6,7,8,11
		Using public transport	1,2,3,6,7,8,11
		Not smoking/reducing smoking	3,4,5,12
		Using natural gas for heating’	1,4,6,9
		Planting trees	1,4,6,8
		Preferring glass products	1,2,4
	Recommended Institutional-Level Strategies	Education	2,4,5,6,7,8,9,10,11,12
		Advertisement/brochure campaign	4,5,6,7,9
		Installation of filters in factory chimneys	5,6,11

The analysis of data obtained from interviews regarding nursing students’ carbon footprint awareness resulted in the identification of two themes, four subthemes, and eighteen codes [23, 24]. Under the theme of Perceived Impacts of Carbon Footprint, the subthemes of Perceived Impact on Human Health and Perceived Impact on Environmental Health were identified. Similarly, under the theme of Carbon Footprint Reduction Strategies, the subthemes of Recommended Individual Strategies and Recommended Institutional Level Strategies were created.

### Sub-Theme 1. Perceived impact on human health

Four codes were identified for perceived impact on human health were identified: ‘affects the respiratory system,’ ‘affects the circulatory system,’ ‘reduces immunity,’ and ‘increases the risk of cancer’ (Table 3). The majority of students stated that the carbon footprint primarily impacts the respiratory system. Additionally, they mentioned that it disrupts the circulatory system, weakens immunity, and increases the likelihood of cancer. Examples of interview excerpts corresponding to the codes identified under Sub-Theme 1 are as follows:

P2: ‘It affects all systems, starting from breathing, respiratory system, immunity, it affects all of them.’
P6: ‘It can cause respiratory system diseases, heart disease and cancer etc.’
P9. ‘I think it can increase some types of cancer.’
P10. ‘It has negative effects on the immune system.’

### Sub-Theme 2. Perceived impact on environmental health

Three codes were identified regarding perceived impact on environmental health were identified: ‘soil pollution caused by plastic products,’ ‘extinction of species,’ and ‘reduction in biodiversity’ (Table 3). The majority of students stated that plastic products harm the environment by polluting the soil. Additionally, they emphasized that the carbon footprint contributes to the extinction of species (plants, animals, etc.) and a decline in biodiversity. Examples of interview excerpts corresponding to the codes identified under sub-theme 2 are as follows:

1. ‘Plastic products are dangerous because they are not easily destroyed in nature.’
2. ‘Since carbon is released into the atmosphere, plant/animal species can be negatively affected.’
3. ‘As the carbon footprint increases, the ecosystem is badly affected. The productivity of plants decreases.’

### Sub-theme 3. Recommended individual strategies

The eight codes for recommended individual strategies were determined as ‘paying attention to waste management’, ‘not littering’, ‘sorting waste’, ‘using public transport’, ‘not smoking/reducing smoking’, ‘using natural gas for heating’, ‘planting trees’ and ‘preferring glass products’ (Table 3). All of the students stated that they paid attention to waste management as an individual measure to reduce carbon footprint. In addition, among the individual measures taken by the students, they stated that they mostly showed behaviours such as not throwing garbage on the ground, separating waste and throwing it in the garbage, and taking care to use public transport. In addition, they also stated that they took measures to reduce their carbon footprint by reducing or not smoking, planting trees and preferring glass products instead of plastic. Some interview examples for the codes determined in sub-theme 3 are as follows;

P3: ‘We throw what we use for the patient into the appropriate medical waste bin. We pay attention to waste management.’
P4: ‘I pay attention not to throw rubbish on the ground. We plant saplings in the garden in our own home. I use glass products instead of plastic.’
P7: ‘I actively use the recycling bin. I pay attention to use public transport.’
P8: ‘I separate the wastes and throw them in the bin. We should get people used to planting saplings.’
P9: ‘I do not throw rubbish on the ground. We switch from coal to natural gas for heating. We use solar energy panels.’
P12: ‘I do not smoke. I warn my friends who smoke not to throw cigarette butts.’

### Sub-theme 4. Recommended institutional level strategies

Three codes for the recommended institutional level strategies were identified as ‘education’, ‘advertisement/brochure campaign’ and ‘installation of filters in factory chimneys’ (Table 3). Students stated that organisations should make plans especially on education and inform the public about carbon footprint, waste management and precautions. They also stated that public awareness should be raised through actions such as advertising campaigns and brochure distribution. In addition, students think that air pollution should be prevented by installing filters on the chimneys of factories. Some interview examples for the codes determined in sub-theme 4 are as follows;

P4: ‘Training can be given in family health centres. Posters can be pasted on billboards.’
P5: ‘Awareness of environmental pollution can be created by creating brochures about environmental pollution.’
P9: ‘I think the municipality should provide training to the public.’
P11: ‘Sanctions can be imposed on factory chimneys to install filters.’

## Discussion

Nursing students’ developing positive attitudes towards carbon footprint may contribute to the spread of environmentally friendly policies in health services in the future. One of the important outcomes of undergraduate education in nursing is to create environmental health awareness [25]. However, it is reported that undergraduate students have insufficient knowledge about environmental health [26]. Increasing the awareness of nursing students on this issue is critical to achieve sustainability goals in health services [17, 25]. Based on this importance, in this study, students’ awareness and recommendations regarding the carbon footprint affecting environmental health were discussed in the light of the literature.

Carbon footprint is a critical aspect of climate change as it measures the impact of human activities on the environment and plays an important role in sustainable development [27]. Considering that the environment is an important determinant of human health and that environmental health and public health are interconnected, carbon footprint has serious negative effects on human health worldwide. In the study, students mostly stated that carbon footprint negatively affects the respiratory system and also affects the circulatory system, decreases immunity and increases the possibility of developing cancer. The negative aspects of the effects of carbon footprint on human health indicated by the students reveal the strong relationship between environmental pollution and health [28]. Students frequently mentioned the effects especially on the respiratory system, which supported the studies in the literature indicating that air pollution and carbon emissions cause asthma, COPD and other respiratory diseases [29, 30]. In parallel with the research results, Okanlı and Demir stated that carbon footprint causes respiratory diseases [31]. Similarly, in a study conducted with nursing students, 73% of nursing students stated that climate change causes cancer and 51.4% stated that it causes respiratory diseases [32]. In addition, findings such as effects on the circulatory system, weakening of the immune system and increase in cancer risk reveal the long-term damages of environmental toxins related to carbon footprint on human health [33, 34]. These results support the need for more effective integration of environmental health issues into the nursing curriculum.

Students stated the effects of carbon footprint on environmental health as ‘plastic products polluting the soil’, ‘not littering’, ‘sorting waste’ and ‘using public transport’. Regarding environmental health, it was also observed that students developed awareness about the environmental effects of carbon footprint. Stating that plastic products do not disappear in nature in a short time, the students showed that they were aware of the long-term effects of environmental pollution. The serious threats of plastic pollution on the ecosystem and human health are also emphasised in the literature [35]. In addition, the fact that students took individual measures such as ‘not littering’, ‘sorting waste’ and ‘using public transport’ shows that environmental awareness is reflected in daily life practices. This finding supports the literature that individual awareness and habits play an important role in environmental sustainability [36]. It is also seen that students exhibit behaviours to reduce their carbon footprint such as ‘using public transport’. This shows that they consciously evaluate their transport preferences at the point of reducing carbon emissions. In the literature, it is widely stated that the use of public transport reduces the negative effects on the environment compared to individual car use [37]. In this context, it can be said that the education programs for students on environmental health are effective [25]. As a result, it is thought that students’ awareness of environmental health can be associated with the knowledge and values they gained during nursing education.

Another important result of our research is that students develop suggestions for individual and social strategies and measures to reduce carbon footprint. Suggestions such as ‘paying attention to waste management’ and ‘installing filters on factory chimneys’ reveal the importance of raising environmental awareness. In the literature, it has been stated that the effective implementation of waste management plays an important role in reducing carbon emissions [38, 39]. In the literature, it is emphasised that filtering and other green technology applications in industrial facilities significantly reduce carbon emissions [40]. In addition, it was observed that students adopted a conscious approach to reducing their carbon footprint by exhibiting behaviours such as using public transport. The suggestion of ‘advertisement/brochure campaign’ reveals that students drew attention to the importance of informing and educating the society in order to increase awareness of carbon footprint. It is supported by previous studies that efforts to raise social awareness increase individual and social participation in environmental problems [41]. This suggestion of the students shows the importance of disseminating environmental education. These results suggest that increasing nursing students’ sensitivity to environmental problems will contribute to social sustainability goals. The nursing profession has a strategic importance in public health and environmental sustainability. Implementation of the students’ suggestions can increase not only individual awareness but also social awareness and promote environmentally friendly policies.

## Conclusion

In line with the results of this study, it was concluded that carbon footprint should be considered as a public health problem and nurses should take an active role in this issue. In order to increase the awareness of nursing students, it is important to integrate environmental awareness and public health issues more comprehensively into the curriculum. In addition, it is thought that practices such as organising training programmes and preparing informative brochures and posters to raise public awareness may be effective. In this direction, conducting more comprehensive, multicentre and long-term studies in different populations as well as nursing students, taking necessary precautions about climate change and passing sustainable environmental awareness to different layers of society and leaving a more livable environment to future generations can be contributed. The nursing profession has a leading position in public health. The dissemination of programmes aimed at raising awareness at both individual and institutional levels can support the creation and implementation of environmentally friendly health policies.

## Data Availability

Datasets are available from the corresponding author upon reasonable request.
